# (*E*)-2-(4-Bromo­benzyl­idene)indan-1-one

**DOI:** 10.1107/S1600536811031746

**Published:** 2011-08-11

**Authors:** Mohamed Ashraf Ali, Rusli Ismail, Tan Soo Choon, Wan-Sin Loh, Hoong-Kun Fun

**Affiliations:** a Institute for Research in Molecular Medicine, Universiti Sains Malaysia, 11800 USM, Penang, Malaysia; b X-ray Crystallography Unit, School of Physics, Universiti Sains Malaysia, 11800 USM, Penang, Malaysia

## Abstract

In the title compound, C_16_H_11_BrO, the dihydro­indene ring system is approximately planar, with a maximum deviation of 0.008 (2) Å. The mean plane of this ring system forms a dihedral angle of 3.73 (11)°, with the bromo-substituted benzene ring. In the crystal, weak inter­molecular C—H⋯O hydrogen bonds link the mol­ecules into sheets parallel to the *ab* plane and further stabilization is provided by weak C—H⋯π inter­actions involving the bromo-substituted benzene rings.

## Related literature

For background information on indanones, see: Schumann *et al.* (2001 ▶); Herzog *et al.* (2002 ▶); Sato (1999 ▶); Leoni *et al.* (2000 ▶); Sugimoto (1999 ▶); Beukes *et al.* (1998) ▶. For closely related structures, see: Ali *et al.* (2010 ▶, 2011 ▶).
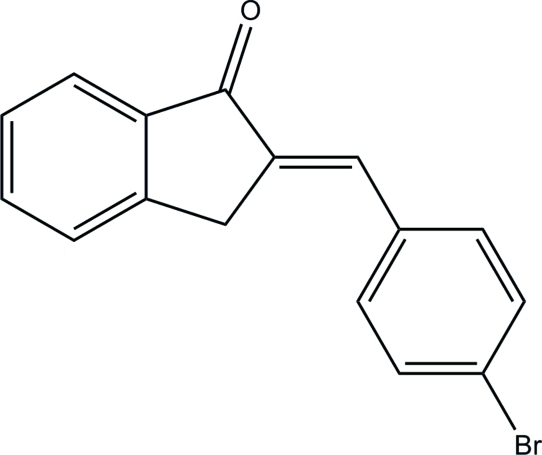


## Experimental

### 

#### Crystal data

C_16_H_11_BrO
*M* *_r_* = 299.16
Monoclinic, 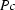

*a* = 6.1933 (7) Å
*b* = 4.7441 (5) Å
*c* = 21.8572 (19) Å
β = 99.108 (3)°
*V* = 634.10 (11) Å^3^
*Z* = 2
Mo *K*α radiation
μ = 3.23 mm^−1^
*T* = 297 K
0.35 × 0.16 × 0.06 mm

#### Data collection

Bruker SMART APEXII DUO CCD area-detector diffractometer
Absorption correction: multi-scan (*SADABS*; Bruker, 2009 ▶) *T*_min_ = 0.398, *T*_max_ = 0.820
6671 measured reflections
2884 independent reflections
2314 reflections with *I* > 2σ(*I*)
*R*_int_ = 0.023

#### Refinement

*R*[*F* ^2^ > 2σ(*F* ^2^)] = 0.027
*wR*(*F* ^2^) = 0.057
*S* = 1.00
2884 reflections
164 parameters
2 restraints
H-atom parameters constrained
Δρ_max_ = 0.25 e Å^−3^
Δρ_min_ = −0.15 e Å^−3^
Absolute structure: Flack (1983 ▶) 1189 Friedel pairs
Flack parameter: 0.00 (6)

### 

Data collection: *APEX2* (Bruker, 2009 ▶); cell refinement: *SAINT* (Bruker, 2009 ▶); data reduction: *SAINT*; program(s) used to solve structure: *SHELXTL* (Sheldrick, 2008 ▶); program(s) used to refine structure: *SHELXTL*; molecular graphics: *SHELXTL*; software used to prepare material for publication: *SHELXTL* and *PLATON* (Spek, 2009 ▶).

## Supplementary Material

Crystal structure: contains datablock(s) global, I. DOI: 10.1107/S1600536811031746/lh5300sup1.cif

Structure factors: contains datablock(s) I. DOI: 10.1107/S1600536811031746/lh5300Isup2.hkl

Supplementary material file. DOI: 10.1107/S1600536811031746/lh5300Isup3.cml

Additional supplementary materials:  crystallographic information; 3D view; checkCIF report

## Figures and Tables

**Table 1:** Hydrogen-bond geometry (Å, °) *Cg*1 is the centroid of the C10–C15 ring.

*D*—H⋯*A*	*D*—H	H⋯*A*	*D*⋯*A*	*D*—H⋯*A*
C4—H4*A*⋯O1^i^	0.93	2.56	3.199 (3)	126
C9—H9*B*⋯O1^ii^	0.97	2.38	3.256 (3)	149
C9—H9*A*⋯*Cg*1^iii^	0.97	2.68	3.528 (3)	147

Symmetry codes: (i) 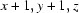; (ii) 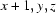; (iii) 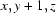.

**Fig. 1.**
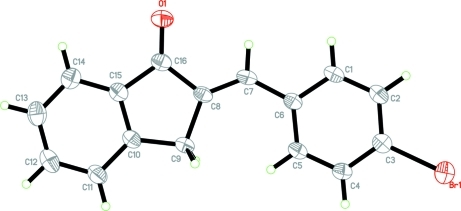
The molecular structure of the title compound, showing 30% probability displacement ellipsoids.

**Fig. 2.**
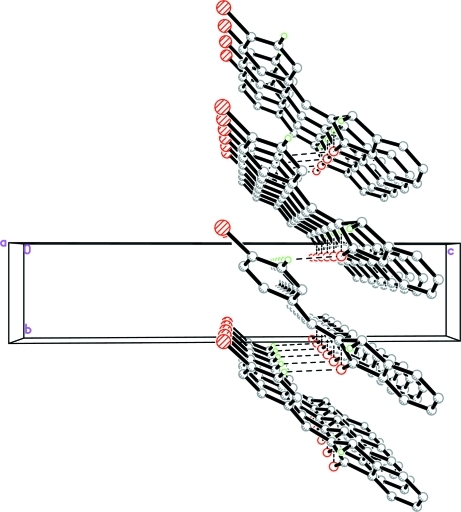
The crystal packing of the title compound, viewed along the a axis. H atoms not involved in the intermolecular interactions (dashed lines) have been omitted for clarity.

## supplementary crystallographic information

### Comment

Indanones and related compounds are important bioactive molecules. These compounds have been studied for various biological activities including cancer and alzheimer's type of diseases. Indanones are also used as drug intermediates, ligands of olefinic polymerisation catalysts and discotic liquid crystals (Schumann *et al.*, 2001; Herzog *et al.*, 2002; Sato, 1999). Another indanone analogue donepezil hydrochloride has been approved by US-FDA for the treatment of mild to moderate alzheimer's disease. This drug acts as an AChE (Acetylcholinesterase) inhibitor and some other indanones have been isolated from natural products. Being such a useful moiety, several synthetic strategies have also been developed for their synthesis (Leoni *et al.*, 2000; Sugimoto, 1999; Beukes *et al.*, 1998). They are very useful intermediates for the synthesis of five and six membered heterocyclic compounds. Dihydroindene derivatives exhibit diverse pharmacological activities. Chemistry of dihydroindene has been recognized as a significant field of study.

In the title compound (Fig. 1), the dihydroindene ring system (C8–C16) is approximately planar, with a maximum deviation of 0.008 (2) Å for atom C15. This ring system is almost coplanar with the benzene ring (C1–C6), with a dihedral angle of 3.73 (11)°. Bond lengths and angles are within the normal ranges and are comparable to those in the related crystal structures (Ali *et al.*, 2010, 2011).

In the crystal (Fig. 2), intermolecular C4—H4A···O1^i^ and C9—H9B···O1^ii^ hydrogen bonds (Table 1) link the molecules into sheets parallel to the *ab* plane (Fig. 2) and further stabilization is provided by C—H···π^iii^ interactions, involving the centroids of benzene rings (C10–C15; *Cg*1).

### Experimental

A mixture of 2,3-dihydro-1*H*-indene-1-one (0.001 mmol) and 4-bromobenzaldehyde (0.001 mmol) was dissolved in methanol (10 mL) and to this mixture was added 30% sodium hydroxide solution (5ml). The mixture was stirred for 5 h. After completion of the reaction, as evident from TLC, the mixture was poured into crushed ice, then neutralized with concentrated HCl. The precipitated solid was filtered, washed with water and recrystallised from ethanol to yield the title compound as light yellow crystals.

### Refinement

All H atoms were positioned geometrically and refined using a riding model with *U*_iso_(H) = 1.2 *U*_eq_(C) [C–H = 0.93–0.97 Å]. The crystal is a twin with twin law, -1 0 0, 0 1 0, 0 0 -1 and BASF = 0.558 (7).

### Crystal data

**Table:**

C_16_H_11_BrO	*F*(000) = 300
*M**_r_* = 299.16	*D*_x_ = 1.567 Mg m^−^^3^
Monoclinic, *P**c*	Mo *K*α radiation, λ = 0.71073 Å
Hall symbol: P -2yc	Cell parameters from 2130 reflections
*a* = 6.1933 (7) Å	θ = 3.3–24.6°
*b* = 4.7441 (5) Å	µ = 3.23 mm^−^^1^
*c* = 21.8572 (19) Å	*T* = 297 K
β = 99.108 (3)°	Plate, colourless
*V* = 634.10 (11) Å^3^	0.35 × 0.16 × 0.06 mm
*Z* = 2	

### Data collection

**Table:**

Bruker SMART APEXII DUO CCD area-detector diffractometer	2884 independent reflections
Radiation source: fine-focus sealed tube	2314 reflections with *I* > 2σ(*I*)
graphite	*R*_int_ = 0.023
φ and ω scans	θ_max_ = 29.0°, θ_min_ = 1.9°
Absorption correction: multi-scan (*SADABS*; Bruker, 2009)	*h* = −8→8
*T*_min_ = 0.398, *T*_max_ = 0.820	*k* = −6→6
6671 measured reflections	*l* = −29→29

### Refinement

**Table:**

Refinement on *F*^2^	Secondary atom site location: difference Fourier map
Least-squares matrix: full	Hydrogen site location: inferred from neighbouring sites
*R*[*F*^2^ > 2σ(*F*^2^)] = 0.027	H-atom parameters constrained
*wR*(*F*^2^) = 0.057	*w* = 1/[σ^2^(*F*_o_^2^) + (0.0281*P*)^2^] where *P* = (*F*_o_^2^ + 2*F*_c_^2^)/3
*S* = 1.00	(Δ/σ)_max_ < 0.001
2884 reflections	Δρ_max_ = 0.25 e Å^−^^3^
164 parameters	Δρ_min_ = −0.15 e Å^−^^3^
2 restraints	Absolute structure: Flack (1983) 1189 Friedel pairs
Primary atom site location: structure-invariant direct methods	Flack parameter: 0.00 (6)

### Special details

**Table:**

Geometry. All esds (except the esd in the dihedral angle between two l.s. planes) are estimated using the full covariance matrix. The cell esds are taken into account individually in the estimation of esds in distances, angles and torsion angles; correlations between esds in cell parameters are only used when they are defined by crystal symmetry. An approximate (isotropic) treatment of cell esds is used for estimating esds involving l.s. planes.
Refinement. Refinement of F^2^ against ALL reflections. The weighted R-factor wR and goodness of fit S are based on F^2^, conventional R-factors R are based on F, with F set to zero for negative F^2^. The threshold expression of F^2^ > 2sigma(F^2^) is used only for calculating R-factors(gt) etc. and is not relevant to the choice of reflections for refinement. R-factors based on F^2^ are statistically about twice as large as those based on F, and R- factors based on ALL data will be even larger.

### Fractional atomic coordinates and isotropic or equivalent isotropic displacement parameters (Å^2^)

**Table:**

	*x*	*y*	*z*	*U*_iso_*/*U*_eq_	
Br1	1.082380 (15)	1.59421 (5)	0.528270 (11)	0.06352 (10)	
O1	0.1862 (3)	0.3615 (4)	0.29957 (9)	0.0621 (5)	
C1	0.5691 (4)	1.0857 (5)	0.45115 (11)	0.0475 (6)	
H1A	0.4363	1.0274	0.4617	0.057*	
C2	0.6967 (4)	1.2730 (6)	0.48919 (11)	0.0508 (6)	
H2A	0.6505	1.3426	0.5247	0.061*	
C3	0.8943 (4)	1.3551 (5)	0.47354 (11)	0.0469 (5)	
C4	0.9614 (4)	1.2637 (6)	0.41993 (11)	0.0478 (6)	
H4A	1.0926	1.3275	0.4093	0.057*	
C5	0.8327 (4)	1.0764 (6)	0.38194 (11)	0.0466 (6)	
H5A	0.8784	1.0126	0.3458	0.056*	
C6	0.6340 (4)	0.9814 (6)	0.39723 (11)	0.0409 (5)	
C7	0.4929 (3)	0.7746 (6)	0.36006 (10)	0.0438 (5)	
H7A	0.3638	0.7333	0.3749	0.053*	
C8	0.5191 (4)	0.6371 (5)	0.30879 (11)	0.0399 (5)	
C9	0.7021 (4)	0.6449 (5)	0.26989 (10)	0.0414 (6)	
H9A	0.7175	0.8319	0.2532	0.050*	
H9B	0.8403	0.5885	0.2941	0.050*	
C10	0.6285 (4)	0.4365 (5)	0.21908 (12)	0.0415 (6)	
C11	0.7351 (5)	0.3606 (6)	0.17000 (12)	0.0541 (6)	
H11A	0.8682	0.4418	0.1654	0.065*	
C12	0.6374 (5)	0.1604 (6)	0.12821 (13)	0.0635 (7)	
H12A	0.7059	0.1078	0.0951	0.076*	
C13	0.4405 (6)	0.0381 (7)	0.13496 (14)	0.0609 (7)	
H13A	0.3791	−0.0966	0.1065	0.073*	
C14	0.3334 (4)	0.1125 (6)	0.18326 (12)	0.0529 (6)	
H14A	0.2006	0.0305	0.1879	0.063*	
C15	0.4308 (4)	0.3145 (5)	0.22484 (10)	0.0445 (5)	
C16	0.3531 (4)	0.4282 (5)	0.28031 (12)	0.0450 (6)	

### Atomic displacement parameters (Å^2^)

**Table:**

	*U*^11^	*U*^22^	*U*^33^	*U*^12^	*U*^13^	*U*^23^
Br1	0.06614 (16)	0.06279 (16)	0.06060 (15)	−0.0043 (2)	0.00682 (10)	−0.00762 (18)
O1	0.0404 (9)	0.0778 (14)	0.0716 (12)	−0.0037 (9)	0.0194 (8)	−0.0069 (10)
C1	0.0478 (12)	0.0513 (15)	0.0479 (12)	0.0046 (13)	0.0210 (10)	0.0048 (12)
C2	0.0597 (15)	0.0527 (16)	0.0439 (12)	0.0045 (13)	0.0204 (11)	0.0013 (11)
C3	0.0507 (12)	0.0432 (14)	0.0467 (12)	0.0087 (11)	0.0078 (10)	0.0069 (10)
C4	0.0453 (12)	0.0522 (16)	0.0481 (12)	−0.0004 (12)	0.0138 (9)	0.0055 (12)
C5	0.0465 (12)	0.0543 (17)	0.0421 (12)	0.0031 (13)	0.0171 (9)	0.0028 (11)
C6	0.0414 (11)	0.0427 (12)	0.0402 (12)	0.0073 (11)	0.0116 (10)	0.0060 (11)
C7	0.0361 (10)	0.0533 (15)	0.0451 (12)	0.0051 (11)	0.0157 (9)	0.0090 (11)
C8	0.0346 (10)	0.0449 (14)	0.0417 (12)	0.0098 (10)	0.0109 (10)	0.0070 (10)
C9	0.0388 (12)	0.0442 (15)	0.0438 (12)	0.0063 (11)	0.0143 (10)	0.0058 (10)
C10	0.0450 (12)	0.0384 (14)	0.0425 (13)	0.0117 (11)	0.0111 (10)	0.0062 (10)
C11	0.0613 (14)	0.0538 (16)	0.0519 (13)	0.0039 (13)	0.0232 (12)	−0.0010 (12)
C12	0.083 (2)	0.0614 (18)	0.0494 (14)	0.0138 (16)	0.0208 (13)	−0.0018 (13)
C13	0.073 (2)	0.0552 (17)	0.0513 (16)	0.0106 (17)	−0.0003 (13)	−0.0036 (13)
C14	0.0481 (13)	0.0514 (16)	0.0558 (14)	0.0044 (13)	−0.0026 (11)	0.0017 (12)
C15	0.0435 (11)	0.0442 (13)	0.0455 (12)	0.0102 (11)	0.0065 (9)	0.0051 (10)
C16	0.0360 (12)	0.0512 (16)	0.0480 (13)	0.0111 (11)	0.0073 (10)	0.0076 (11)

### Geometric parameters (Å, °)

**Table:**

Br1—C3	1.906 (3)	C8—C9	1.521 (3)
O1—C16	1.219 (3)	C9—C10	1.503 (4)
C1—C2	1.378 (4)	C9—H9A	0.9700
C1—C6	1.395 (3)	C9—H9B	0.9700
C1—H1A	0.9300	C10—C15	1.377 (4)
C2—C3	1.378 (4)	C10—C11	1.393 (4)
C2—H2A	0.9300	C11—C12	1.388 (4)
C3—C4	1.374 (3)	C11—H11A	0.9300
C4—C5	1.380 (4)	C12—C13	1.379 (5)
C4—H4A	0.9300	C12—H12A	0.9300
C5—C6	1.400 (3)	C13—C14	1.378 (4)
C5—H5A	0.9300	C13—H13A	0.9300
C6—C7	1.470 (4)	C14—C15	1.391 (4)
C7—C8	1.329 (3)	C14—H14A	0.9300
C7—H7A	0.9300	C15—C16	1.476 (3)
C8—C16	1.491 (4)		
			
C2—C1—C6	121.6 (2)	C8—C9—H9A	111.1
C2—C1—H1A	119.2	C10—C9—H9B	111.1
C6—C1—H1A	119.2	C8—C9—H9B	111.1
C1—C2—C3	118.7 (2)	H9A—C9—H9B	109.1
C1—C2—H2A	120.7	C15—C10—C11	119.9 (2)
C3—C2—H2A	120.7	C15—C10—C9	112.3 (2)
C4—C3—C2	121.5 (2)	C11—C10—C9	127.9 (2)
C4—C3—Br1	119.07 (19)	C12—C11—C10	118.2 (3)
C2—C3—Br1	119.40 (19)	C12—C11—H11A	120.9
C3—C4—C5	119.5 (2)	C10—C11—H11A	120.9
C3—C4—H4A	120.3	C13—C12—C11	121.2 (3)
C5—C4—H4A	120.3	C13—C12—H12A	119.4
C4—C5—C6	120.6 (2)	C11—C12—H12A	119.4
C4—C5—H5A	119.7	C14—C13—C12	121.0 (3)
C6—C5—H5A	119.7	C14—C13—H13A	119.5
C1—C6—C5	118.0 (2)	C12—C13—H13A	119.5
C1—C6—C7	118.6 (2)	C13—C14—C15	117.7 (3)
C5—C6—C7	123.4 (2)	C13—C14—H14A	121.1
C8—C7—C6	130.7 (2)	C15—C14—H14A	121.1
C8—C7—H7A	114.6	C10—C15—C14	122.0 (2)
C6—C7—H7A	114.6	C10—C15—C16	109.4 (2)
C7—C8—C16	120.7 (2)	C14—C15—C16	128.7 (2)
C7—C8—C9	131.4 (2)	O1—C16—C15	126.4 (2)
C16—C8—C9	108.0 (2)	O1—C16—C8	126.5 (2)
C10—C9—C8	103.4 (2)	C15—C16—C8	107.0 (2)
C10—C9—H9A	111.1		
			
C6—C1—C2—C3	−0.8 (4)	C9—C10—C11—C12	179.6 (3)
C1—C2—C3—C4	2.7 (4)	C10—C11—C12—C13	−0.3 (4)
C1—C2—C3—Br1	−175.86 (18)	C11—C12—C13—C14	0.5 (5)
C2—C3—C4—C5	−2.6 (4)	C12—C13—C14—C15	−0.1 (4)
Br1—C3—C4—C5	175.93 (18)	C11—C10—C15—C14	0.8 (4)
C3—C4—C5—C6	0.7 (4)	C9—C10—C15—C14	−179.1 (2)
C2—C1—C6—C5	−1.1 (4)	C11—C10—C15—C16	179.7 (2)
C2—C1—C6—C7	177.9 (2)	C9—C10—C15—C16	−0.3 (3)
C4—C5—C6—C1	1.2 (4)	C13—C14—C15—C10	−0.6 (4)
C4—C5—C6—C7	−177.8 (2)	C13—C14—C15—C16	−179.2 (3)
C1—C6—C7—C8	−177.5 (3)	C10—C15—C16—O1	−178.5 (3)
C5—C6—C7—C8	1.5 (4)	C14—C15—C16—O1	0.3 (4)
C6—C7—C8—C16	178.2 (2)	C10—C15—C16—C8	0.7 (3)
C6—C7—C8—C9	−0.6 (5)	C14—C15—C16—C8	179.5 (2)
C7—C8—C9—C10	179.5 (3)	C7—C8—C16—O1	−0.7 (4)
C16—C8—C9—C10	0.7 (2)	C9—C8—C16—O1	178.3 (3)
C8—C9—C10—C15	−0.2 (3)	C7—C8—C16—C15	−179.8 (2)
C8—C9—C10—C11	179.8 (3)	C9—C8—C16—C15	−0.9 (2)
C15—C10—C11—C12	−0.4 (4)		

### Hydrogen-bond geometry (Å, °)

**Table:**

Cg1 is the centroid of the C10–C15 ring.

**Table:**

*D*—H···*A*	*D*—H	H···*A*	*D*···*A*	*D*—H···*A*
C4—H4A···O1^i^	0.93	2.56	3.199 (3)	126
C9—H9B···O1^ii^	0.97	2.38	3.256 (3)	149
C9—H9A···Cg1^iii^	0.97	2.68	3.528 (3)	147

Symmetry codes: (i) *x*+1, *y*+1, *z*; (ii) *x*+1, *y*, *z*; (iii) *x*, *y*+1, *z*.
